# Exploring Endotypes in Chronic Rhinosinusitis (ExpRess): Protocol for a cohort study

**DOI:** 10.1371/journal.pone.0289407

**Published:** 2023-08-02

**Authors:** Shyam Ajay Gokani, Matthew Jefferson, Jelena Gavrilovic, Allan Clark, Falk Hildebrand, Tom Wileman, Claire Hopkins, Carl Philpott

**Affiliations:** 1 Rhinology and ENT Research Group, Norwich Medical School, University of East Anglia, Norwich, United Kingdom; 2 Norwich Medical School, University of East Anglia, Norwich, United Kingdom; 3 Quadram Institute Bioscience, Norwich, United Kingdom; 4 Department of Otolaryngology, Guy’s and St Thomas’ NHS Foundation Trust, London, United Kingdom; University Putra Malaysia, MALAYSIA

## Abstract

**Background:**

Chronic Rhinosinusitis (CRS) affects approximately 1 in 10 UK adults and impacts quality of life quality of life significantly. Response to treatment may be driven by individual CRS endotypes and therefore work to delineate biomarker clusters that may separate responders from non-responders is needed. The ongoing MACRO three-arm parallel-group trial randomises adult CRS patients to endoscopic sinus surgery, macrolide therapy or placebo.

**Aim:**

This study aims to correlate CRS endotypes with clinical parameters from the ongoing MACRO trial, including olfactory function and outcomes in terms of response to treatment using core biomarkers sets.

**Methods:**

Adult CRS patients enrolled into the MACRO trial will be recruited from participating UK otorhinolaryngology departments. Nasal tissue samples and swabs will be obtained in theatre or clinic from patients randomised to all three trial arms. Nasal tissue will be analysed with multiplex electrochemiluminescence for 32 cytokines including IL-5, IL-13, IgE and periostin. Bacterial swabs will be analysed using illumina miSeq 16S amplicon sequencing. Mean expression for each biomarker will be reported for treatment responder and non-responder groups. Correlation of biomarkers with MACRO trial outcome data such as endoscopic evaluation scores and quality-of-life improvement scores will be reported.

**Discussion:**

Defining clear endotypes in CRS will contribute to refining patient pathways for the efficient use of clinical resources. This work may lay the groundwork for future studies to predict which patients might respond to medical or surgical therapy.

## Introduction

Chronic Rhinosinusitis (CRS) affects one in ten adults in the United Kingdom (UK) and impacts significantly on their quality of life [1]. Symptoms include a blocked and runny nose, loss of smell, facial pain, tiredness and worsening of breathing problems, such as asthma. Approximately 1% of UK adults receive treatment for CRS from their general practitioner (GP) each year, with a median of four GP visits. Traditionally, CRS has been dichotomised to two main phenotypes, based on endoscopic appearance of with (CRSwNP) or without nasal polyps (CRSsNP). Several biomarkers have been correlated to CRS phenotypes, such as nasal interleukin-5 (IL-5), IL-13 and periostin to CRSwNP [2, 3]. Furthermore, sinonasal bacteria such as *Staphylococcus aureus* have been implicated in the pathogenesis of CRS and treatments such as macrolide antibiotics have been shown to have immunomodulatory effects in selected patient groups [4].

More recently, a number of distinct CRS endotypes have been described, with incomplete overlap with the phenotypes above. Response to treatment may be driven by individual CRS endotypes [5] and therefore work to delineate key biomarker clusters that may separate responders from non-responders is needed. The relationship between disease severity, biomarkers and microbiome profiles is still unclear.

The ongoing MACRO three-arm parallel-group trial aims to establish best practice for the management of adults with CRS [6]. Recruitment began in autumn 2018 and to date over 350 patients have been randomised across 17 UK sites to receive either:

1. intranasal corticosteroids with saline irrigation plus endoscopic sinus surgery (ESS),
2. intranasal corticosteroids with saline irrigation plus clarithromycin (250 mg twice daily for 2 weeks then once daily for 10 weeks), or
3. intranasal corticosteroids with saline irrigation plus placebo.

The primary outcome measure for the MACRO trial is the sino-nasal outcome test-22 (SNOT-22) score at 6 months after randomisation. Secondary outcomes include the endoscopic Lund–Kennedy score, olfactory function measured using Sniffin’ Sticks, and health-care resource use. The parallel ExpRess study involves collection of nasal tissue samples and bacterial swabs from MACRO trial participants. The ExpRess study aims to correlate CRS endotypes with clinical parameters from the ongoing MACRO trial, including olfactory function and outcomes in terms of response to treatment using core biomarkers sets. This will further refine the biomarker sets that could be utilised in future clinical practice for personalised treatment for CRS.

## Methods

### Objectives

The ExpRess study is a multicentre prospective cohort study of adult patients with CRS recruited from at least five UK hospitals in parallel to the MACRO randomised controlled trial. Recruitment is currently ongoing at James Paget University Hospital, Norfolk and Norwich University Hospital, Guy’s and St Thomas’ NHS Foundation Trust, Imperial College Healthcare NHS Trust and Newcastle upon Tyne Hospitals NHS Foundation Trust. Study objectives include:

1. To quantify the cytokine expression pattern for MACRO trial participants,
2. To determine if cytokine expression varies between treatment responder and non-responder groups,
3. To identify if phenotypic characteristics collected from MACRO patients can be predicted by cytokine profiles,
4. To define the bacterial profiles of MACRO trial participants.

### Participants

All participants are recruited from existing MACRO trial and are subject to the full eligibility criteria detailed in the MACRO protocol [6]. Selected eligibility criteria include:

- Adults aged 18 and over with a minimum of 12 weeks’ history of inflammation of the nose and paranasal sinuses characterised by two or more symptoms, one of which should be either (1) nasal blockage, obstruction or congestion or (2) nasal discharge (anterior or posterior nasal drip). Other symptoms may include facial pain or pressure and reduction or loss of smell.
- Moderate or severe symptoms within the last 3 months with a SNOT-22 score ≥ 20.
- Symptom control not achieved following previous alternative medical therapy, including intranasal corticosteroids, short courses of antibiotics, or saline rinses, and therefore considered eligible for ESS.

Participants will be excluded if they meet any of the following criteria or those detailed in the full MACRO trial protocol:

- Lund–Mackay non-contrast CT scan score < 4.
- Macrolide antibiotic treatment for >3 continuous weeks within the last 12 months.
- ESS in the previous 6 months or visible, open sinus cavities from previous surgery.
- Taking maintenance oral steroids or biologics
- Has received systemic corticosteroids in the last one month.
- Rare or complex sinus conditions, such as CRS secondary to systemic disease (e.g. ciliary dyskinesias or granulomatous diseases) or suspected malignancy.
- Allergic fungal rhinosinusitis confirmed or suspected on CT imaging necessitating immediate surgery.
- Severe asthma (taking high doses of inhaled steroids, i.e. >1.5 mg per day).

All participants will have received nasal endoscopy within 3 months prior to recruitment to confirm CRS phenotype, and a non-contrast CT scan within 18 months prior to recruitment to confirm suitability for ESS.

### Recruitment

Participants will be recruited from January 2019 to September 2023 from participating UK sites. The recruitment target will be 120 patients in total across all three arms of the MACRO trial; equating to approximately 40 participants per arm. An a-priori sample size calculation was not possible due to lack of information around the baseline prevalence and variance of each cytokine included within the study groups and the likely difference between groups.

**Fig 1:**
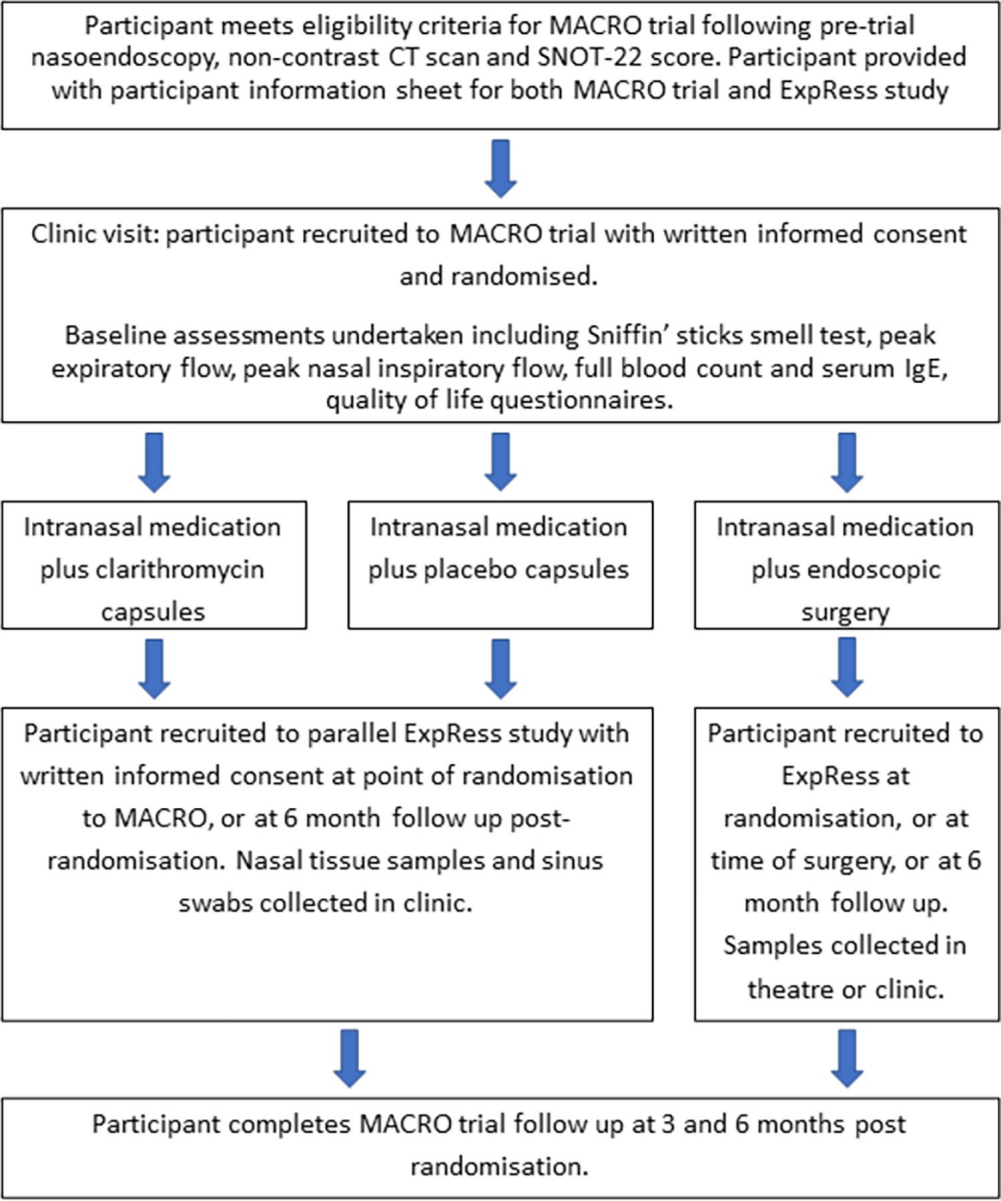
ExpRess study flowchart.

Patients will be approached for recruitment to ExpRess following randomisation to any of the three MACRO trial arms of surgery, macrolide antibiotics or placebo. Patients will be approached in clinic by the principal investigator or the research nurse and will be given a recruitment pack that will include a participant information sheet (S1 File) and consent forms. Following written informed consent, patients will have nasal tissue samples and endoscopically guided nasal swabs collected either at the point of randomisation or at their 6 month follow up visit. Participants randomised to the surgical arm of the MACRO trial are also eligible to have samples collected at the time of surgery instead, which is within 6 weeks of randomisation. This process is outlined in Fig 1.

### Sample collection

Nasal tissue samples are collected by an otorhinolaryngologist either in clinic or in the operating theatre for participants randomised to ESS. Tissue is sampled during nasoendoscopy or ESS as a biopsy from the uncinate process of participants with CRS without nasal polyps (CRSsNP) or from nasal polyps for CRSwNP participants. Participants in clinic receive topical anaesthetic with 5% lidocaine and 0.5% phenylephrine up to five minutes prior to sample collection. Samples are stored in 2ml cryovials at -20°C at the collection site until transport to the Biorepository situated in Norwich Research Park at the University of East Anglia. Transport to the biorepository is assured by the local research teams and is performed in batches in a box filled with ice. After reception at the biorepository, samples are anonymised and stored at -80°C until analysis. Samples are pseudoanonymously linked to the MACRO trial database in Oxford.

Participants will also have endoscopically guided nasal swabs collected using DNA/RNA shield swabs (Cambridge Bioscience, Cambridge, UK). Nasal swabs for each participant will be taken from both the left and right middle meatus at a directional vector of 0–20 degrees in relation to the floor of the nose. Swabs will be stored at room temperature or colder and transported alongside nasal tissue samples to the Norwich Biorepository for storage at -80°C until analysis.

### Laboratory analysis

Nasal tissue samples will be prepared on ice. Protein will be extracted using a small sterile stainless-steel bead within 2.0ml DNA LoBind® tubes (Eppendorf, Hamburg, Germany) which are pre-incubated for 15 minutes on ice. After addition of the nasal tissue sample, the tube will remain on ice for another 15 minutes. According to tissue sample volume, 0.1 to 1ml of buffer solution will be added, consisting of T-PER™ Tissue Protein Extraction Reagent (Thermofisher Scientific, Massachusetts, United States) and cOmplete™ Mini EDTA-free Protease Inhibitor Cocktail (Roche, Basel, Switzerland), in a proportion of 1 tablet for 10 ml of T-PER buffer. Tissue samples will then be placed in a homogeniser at 50Hz for at least 7 minutes and then centrifuged for 10 minutes at 4000rpm at 4°C. The supernatant will then be extracted into clean LoBind® tubes and stored at -80°C prior to analysis. A Pierce™ bicinchoninic acid (BCA) protein assay (Thermofisher Scientific, Massachusetts, United States) will be run on an aliquot of each sample in duplicate to determine protein volumes.

Processed nasal tissue samples will be analysed in duplicate with multiplex detection via electrochemiluminescence, using the MESO QuickPlex SQ 120 (Meso Scale Diagnostics, Maryland, United States). R-PLEX assays for IgE and periostin will be used alongside a custom U-PLEX assay for ENA-78, eotaxin, eotaxin-2, G-CSF, IFN-β, IFN-γ, IL-1β, IL-2, IL-4, IL-5, IL-6, IL-8, IL-9, IL-10, IL-12p70, IL-13, IL-15, IL-17A, IL-22, IL-23, IL17E/IL25, IL-33, MCP-1, MCP-4, MIP-1α, MIP-3α, TNF-α, TSLP, VEGF-A and BAFF. 50–100μg of protein per sample will be used for each assay.

Microbiome analysis for nasal bacteriology swabs would be undertaken in conjunction with a bioinformatics team at the Quadram Institute Bioscience in Norwich. The LotuS2 pipeline developed in this group will be used to analyse these samples, having demonstrated that this pipeline produces more accurate microbiome reconstructions than contemporary pipelines [7]. Due to these samples likely being relatively low bacterial–high human biomass (compared to other environments), special care will be taken to avoid human contaminants using a technique developed in this group [8].

### Outcome measures

The primary outcome measure will be mean expression for each cytokine listed within a core set of biomarkers for treatment responder and non-responder groups. Responders will be defined as participants with a minimum 10-point improvement from their baseline SNOT-22 score at 6 months from randomisation to the MACRO trial. The cytokine data and microbiome profiles will be linked with the MACRO trial data from the Oxford Surgical Intervention Trials Unit following completion of the MACRO trial. MACRO data includes participant demographic information, disease-specific health-related quality of life using the SNOT-22 at 6 months, endoscopic Lund–Kennedy score, Lildholdt polyp grading score, health-related quality of life and quality-adjusted life-years using the SF-12v2 and EQ-5D-5 L questionnaires, olfactory function measured using Sniffin’ Sticks, and health-care resource use recorded using patient questionnaires.

### Statistical analysis

Data will be analysed using RStudio version 1.2.13 software (RStudio, Massachusetts, United States). For continuous variables, results will be expressed as mean and standard deviation. Data distribution will be tested for normality using a Shapiro-Wilk test. If the variables are not normally distributed, the following statistical tests will be used: for dichotomous variables, a chi-square test will be used to determine difference between groups; while, for continuous variables, a Kruskal-Wallis test will be used for between-group comparison. If expression levels of biomarkers are below the detection limit, they will be replaced by the value 0 for analysis purposes. A p-value of <0.05 is considered significant.

For objective 1 the expression of each cytokine will be expressed as mean and standard deviation for participants from each MACRO trial arm. For objective 2 each MACRO trial arm will be analysed separately. Logistic regression analysis will be performed to determine the impact of cytokine expression levels on binary responder status (defined as a minimum 10-point improvement in baselines SNOT-22 score at 6 months). For objective 3 logistic regression analysis will be performed to determine the impact of cytokine expression levels on phenotypic characteristics such as the presence of nasal polyps or raised total serum IgE (>100 IU/ml). For objective 4 microbiome profiles will be reported as mean distribution of bacterial strains. Bacterial profiles will be compared alongside outcomes from the MACRO trial database using univariate and multivariate statistics, associating microbiome profiles to cytokine profiles and treatment success on included patients.

### Timescale

Sample recruitment began in January 2019 and will be completed by September 2023. Sample analysis is due to begin in March 2023. Final results will be available in March 2024 once results from the MACRO trial database are available and can be linked to the biomarker analysis.

### Ethical approval

This study was approved by the NHS HRA East Midlands—Leicester Central Research Ethics Committee under the reference 16/EM/0468 (S2, S3 Files). All participants will provide written informed consent.

## Discussion

Defining clear endotypes in CRS will help to refine patient pathways for the efficient use of medical and surgical resources. This work may lay the groundwork for future studies to predict which patients might respond to medical or surgical therapy. Patients undergoing surgery for CRS in the UK can have treatment costs totalling >£2,000 per year, compared to macrolide antibiotics prescriptions costing <£10/year [9]. In a retrospective study of 88,317 patient with CRS, 46% were prescribed antibiotics for CRS and 26% had more than 10 recorded prescriptions during follow up, suggesting that CRS is a significant target for antibiotic stewardship [10]. Better understanding of the CRS microbiome and biomarker profiles will help to rationalise these prescriptions in primary care and contribute to targeting the global threat of antimicrobial resistance.

The strengths of this study include a large sample size and multicentre approach to recruitment to ensure a diversity of CRS phenotypes is captured. Limitations include the restriction of cytokine targets due to resources available within our institution. However, the most important cytokine targets such as IL-5, IL-13 and periostin have been identified through a thorough literature search by our team and are including within our analysis. An additional limitation is the lack of microbiome data for patients recruited to ExpRess prior to November 2022. Due to a lack of data on the baseline values and variance of all cytokines and microbiome profiles within the study population, formal sample size calculations were not possible. We will therefore liaise closely with recruiting centres and aim to extend the study to other sites to ensure adequate patient numbers for microbiome analysis are achieved.

Any study amendments will be submitted to the Integrated Research Application System for subsequent management by the Research Ethics Committee. Amendments will require approval by the study team and all study documents will be kept updated appropriately. We would aim to publish the results of this study in peer reviewed journals and present nationally and internationally at relevant meetings. Lay summaries for patients and the public will be produced and disseminated through the MACRO trial and University of East Anglia Rhinology and ENT Research Group websites.

## Supporting information

S1 Checklist STROBE checklist. (DOCX)

S1 File ExpRess study participant information sheet v1.7. (DOCX)

S2 File ExpRess study protocol v1.5 (HRA approved). (DOCX)

S3 File Letter of health research authority approval. (PDF)

## References

[1] Gliklich RE, Metson R. The health impact of chronic sinusitis in patients seeking otolaryngologic care. Otolaryngol Head Neck Surg. 1995 Jul;113(1):104–9. doi: 10.1016/S0194-59989570152-4 7603703

[2] Lin YT, Chen WC, Tsai MH, Chen JY, Chien CY, Huang SC. JAK2 Phosphorylation Signals and Their Associated Cytokines Involved in Chronic Rhinosinusitis with Nasal Polyps and Correlated with Disease Severity. Biomolecules. 2021;11(7). doi: 10.3390/biom11071059 34356683 PMC8301971

[3] Maxfield AZ, Landegger LD, Brook CD, Lehmann AE, Campbell AP, Bergmark RW, et al. Periostin as a Biomarker for Nasal Polyps in Chronic Rhinosinusitis. Otolaryngol Head Neck Surg. 2018;158(1):181–6. doi: 10.1177/0194599817737967 29040053

[4] Head K, Chong LY, Piromchai P, Hopkins C, Philpott C, Schilder AGM, et al. Systemic and topical antibiotics for chronic rhinosinusitis. Cochrane Database Syst Rev. 2016 Apr 26;4:CD011994. doi: 10.1002/14651858.CD011994.pub2 27113482 PMC8763400

[5] Morse JC, Shilts MH, Ely KA, Li P, Sheng Q, Huang LC, et al. Patterns of olfactory dysfunction in chronic rhinosinusitis identified by hierarchical cluster analysis and machine learning algorithms. Int Forum Allergy Rhinol. 2019;9(3):255–64. doi: 10.1002/alr.22249 30485725 PMC6397071

[6] Philpott C, le Conte S, Beard D, Cook J, Sones W, Morris S, et al. Clarithromycin and endoscopic sinus surgery for adults with chronic rhinosinusitis with and without nasal polyps: study protocol for the MACRO randomised controlled trial. Trials. 2019 Apr 29;20(1):246. doi: 10.1186/s13063-019-3314-7 31036048 PMC6489242

[7] Özkurt E, Fritscher J, Soranzo N, Ng DYK, Davey RP, Bahram M, et al. LotuS2: an ultrafast and highly accurate tool for amplicon sequencing analysis. Microbiome. 2022 Oct 19;10(1):176. doi: 10.1186/s40168-022-01365-1 36258257 PMC9580208

[8] Bedarf JR, Beraza N, Khazneh H, Özkurt E, Baker D, Borger V, et al. Much ado about nothing? Off-target amplification can lead to false-positive bacterial brain microbiome detection in healthy and Parkinson’s disease individuals. Microbiome. 2021 Mar 26;9(1):75. doi: 10.1186/s40168-021-01012-1 33771222 PMC8004470

[9] Clarke CS, Williamson E, Denaxas S, Carpenter JR, Thomas M, Blackshaw H, et al. Observational retrospective study calculating health service costs of patients receiving surgery for chronic rhinosinusitis in England, using linked patient-level primary and secondary care electronic data. BMJ Open. 2022 Feb 8;12(2):e055603. doi: 10.1136/bmjopen-2021-055603 35135774 PMC8830221

[10] Hopkins C, Williamson E, Morris S, Clarke CS, Thomas M, Evans H, et al. Antibiotic usage in chronic rhinosinusitis: analysis of national primary care electronic health records. Rhinology. 2019 Dec 1;57(6):420–9. doi: 10.4193/Rhin19.136 31490466

