# Supplementary material for: Exploring Endotypes in Chronic Rhinosinusitis (ExpRess): Protocol for a cohort study
Source: PLoS One. 2023 Aug 2;18(8):e0289407. doi: 10.1371/journal.pone.0289407 (PMC10395813; doi:10.1371/journal.pone.0289407)
Supplement: S1 File — (DOCX) [file pone.0289407.s002.docx]

# Exploring Endotypes in Chronic Rhinosinusitis

IRAS Number: 202585

Participant Information Form v1.7 20.09.2022

**Project Sponsor:** University of East Anglia

**Chief Investigator:** Carl Philpott

**Co-Investigators:** Claire Hopkins

Shyam Gokani

Tom Wileman

Falk Hildebrand

**Location:** James Paget University Hospital

University of East Anglia and its associated NHS Hospitals

**Dear _________________**

**Introduction**

You are invited to take part in this research project that is investigating the condition chronic rhinosinusitis (CRS). This is because you have been identified as suffering with CRS.

Please take a few moments to read this document that will help you decide whether you wish to take part.

**What is chronic rhinosinusitis?**

CRS is a condition where the lining of the sinuses and also the nose becomes swollen and the resulting symptoms have lasted for at least 3 months. At present, when patients with CRS present to Ear, Nose and Throat doctors, they will typically examine the nose to see whether or not there are any polyps (grape like swellings) sitting in the nasal cavity. This leads to 2 main groupings of CRS with nasal polyps (CRSwNPs) and CRS without nasal polyps (CRSsNPs) – these are known as phenotypes.

**What are endotypes?**

As opposed to phenotypes, which are determined by the physical characteristics of the disease found on examination, endotypes are determined by chemicals produced in the sinus/nasal tissue and mucus. There is increasing evidence that certain groups of patients with CRS have certain patterns of chemicals that they produce and that these patterns will also help to predict which treatments will work best for these specific groups. We believe there are a much larger number of endotypes, as compared to the two basic phenotypes we have described above, but this has not yet been clearly defined and as such current treatment recommendations are largely based on whether or not a patient has polyps present in their nose. In practice, we know that within these two groups, patients can respond very differently to treatment, so by defining the underlying endotypes, we hope to understand why that is. We also wish to determine whether the profile of micro-organisms in the nose (nasal microbiome) of patients is related to their endotype or treatment outcomes.

**What does participation in this research involve?**

As you have CRS and are participating in the MACRO trial and have chosen to undergo endoscopic sinus surgery, we are asking permission to keep remove some of the swollen tissue removed from in your nose/sinuses (such as polyps) for research. If you are having sinus surgery, this is tissue that we would normally get rid of at the end of the operation. In addition to this we would want to capture some of the mucus present in your nose and sinuses. This will be taken via a swab of your nose. All of this tissue will then be transferred to the tissue bank for storage until we are ready to test it in our laboratory. In addition to your permission to use these samples for research, we would also want to match the data collected from you as part of the MACRO trial with the findings in the laboratory. By doing this we hope to be able to learn more about why some patients respond better than others to the same treatments that are commonly used for CRS. We will notify the Oxford Surgical Trials Unit that you are participating in this study but giving them a unique study number that they will enter alongside the data they collect as part of the MACRO trial.

We hope that you will be able to help us but we do of course understand if you do not wish to participate and will fully respect this decision.

**What are the benefits to you of taking part?**

There are no direct benefits to you from taking part in this project. This is scientific research aimed at understanding the biology of the condition so that we will better be able to treat the condition in the future.

**What are the disadvantages or risks of taking part?**

If you are undergoing surgery in the trial, you will be asleep (under anaesthetic) during the procedure, so you will not experience any discomfort from these samples being removed beyond that which you would expect from the surgery itself. If you are receiving medical treatment in the trial, we will use some local anaesthetic to numb your nose before taking a small sample of tissue.

We will be looking at your personal health records relating to your CRS, but will maintain the confidentiality you would expect.

**How will we tell you what we have found?**

This study will form the basis of an academic clinical fellowship for Dr Shyam Gokani, as well as a PhD studentship for Ana Pratas. We have an existing research website that we will use to announce results. We will also contact our patient charity Fifth Sense, so that they can make announcements on their website and their newsletters. We will use this to update participants on the progress of our research and to let you know of any of our findings. We hope to publish any findings of significance in scientific journals. We plan to utilise the initial findings to help further refine our investigations during a national trial of CRS treatment known as the MACRO Programme: Defining best management of adult chronic rhinosinusitis.

**Further information**

**What will happen if I don’t want to take part?**

There will be no impact on any care that you may need.

**How will my information be kept confidential?**

The information about your tissue will be kept in confidential in hospital and university computers. The information will be coded so as to be only available to the research team. The University of East Anglia (UEA) is the sponsor for this study. We will be using information from you and/or your medical records in order to undertake this study and will act as the data controller for this study. This means that we are responsible for looking after your information and using it properly. UEA will keep identifiable information about you 10 years after the study has finished. Your rights to access, change or move your information are limited, as we need to manage your information in specific ways in order for the research to be reliable and accurate. If you withdraw from the study, we will keep the information about you that we have already obtained. To safeguard your rights, we will use the minimum personally-identifiable information possible. You can find out more about how UEA manages Personal Data by contacting UEA Data Protection Team ([dataprotection@uea.ac.uk)](mailto:dataprotection@uea.ac.uk)).

**Who is organising and funding this study?**

This study is organised in Norfolk by a team of ENT surgeons and scientists. It is funded from scientific research grants that have been competitively awarded by the Sir Jules Thorn Charitable Trust and ENT UK.

**Will I be paid for taking part?**

No. We are seeking volunteers to help us with this study. We may be in a position to refund reasonable travel expenses if these are necessarily incurred.

**What will happen to the tissue samples and mucus that I give?**

The tissue sample and swabs will be sent to the Norwich Biorepository and stored in a freezer at -80**°**C**.** We have no immediate plans to undertake any genetic testing but the sample will be stored for 10 years as to be available for future ethically approved research when the DNA can be extracted.

**Who has reviewed the study?**

All research that involves NHS patients or staff, information from NHS medical records or uses NHS premises or facilities has to be approved by an NHS Research Ethics Committee before it goes ahead. This study has been reviewed by the **East Midlands - Leicester Central Research Ethics Committee**. Approval does not guarantee that you will not come to any harm if you take part, however approval means that the Committee is satisfied that your rights will be respected; that any risks have been reduced to a minimum; have been balanced against possible benefits and that you have been given sufficient information on which to make an informed decision.

**What if I am harmed by the study?**

Nothing in this study is expected to cause you any harm. In the event that something does go wrong and you are harmed during the research study there are no special compensation arrangements. If you are harmed and this is due to someone’s negligence then you may have grounds for a legal action for compensation but you may have to pay your legal costs.

**The normal National Health Service complaints mechanisms will still be available to you through:**

{INSERT LOCAL TRUST DETAILS}

**Contact for further information**

If you have any queries or concerns about any aspect of this study, you should ask to speak to the researchers who will do their best to answer your questions. In this situation please don’t hesitate to contact:

{INSERT LOCAL PI DETAILS}

Independent sponsor contact:

Charles ffrench-Constant

Pro-Vice-Chancellor of Norwich Medical School

University of East Anglia

Norwich

NR4 7LT
