# Supplementary material for: Exploring Endotypes in Chronic Rhinosinusitis (ExpRess): Protocol for a cohort study
Source: PLoS One. 2023 Aug 2;18(8):e0289407. doi: 10.1371/journal.pone.0289407 (PMC10395813; doi:10.1371/journal.pone.0289407)
Supplement: S2 File — (DOCX) [file pone.0289407.s003.docx]

Exploring endotypes in Chronic Rhinosinusitis (ExpRess): Study Protocol

Chief Investigator: Prof. Carl Philpott

Research Team: Prof. Claire Hopkins, Prof. Tom Wileman, Dr. Falk Hildebrand, Dr. Jelena Gavrilovic, Allan Clark, Ana Pratas, Shyam Gokani

# Background

Chronic rhinosinusitis (CRS) represents a common source of ill health; 11% of UK adults report CRS symptoms in a worldwide population study^1^. Symptoms include nasal obstruction, nasal discharge, facial pain, anosmia and sleep disturbance with a major impact on quality of life. The European Position Paper on Rhinosinusitis and Nasal Polyps (EPOS)^2^ has now defined rhinosinusitis the condition on clinical grounds based on the presence of these characteristic symptoms, combined with objective evidence of mucosal inflammation and/or radiological findings. CRS is divided into those with polyps (CRSwNPs) and without polyps (CRSsNPs). This division has been broadly based up the pathophysiological themes within these two main subgroups, either with a predominantly eosinophilic or neutrophilic inflammatory infiltrate respectively but this is very rudimentary. CRS endotypes represent a subtype of the condition defined by a distinct functional or pathobiological mechanism. Clinically practice is variable both in terms of medical and surgical treatment but there is increasing evidence that different CRS endotypes can be characterised by differences in responsiveness to different treatments, including intranasal corticosteroids, biological agents and Manuka honey^3^. It is likely that these differences reflect differing pathophysiology between cases and are based on specific cytokine signatures^4^. Moving away from the concept of CRS as a single disease entity has the potential for more effective treatment and better patient outcomes. Sinonasal bacteria such as Staphylococcus aureus have been implicated in the pathogenesis of CRS and treatments such as macrolide antibiotics have been shown to have immunomodulatory benefits in selected patient groups. The MACRO Programme, “Defining best Management in Adult Chronic RhinOsinusitis”, funded by NIHR at £3.2 million for 7 years commenced in November 2016 and the clinical trial (workstream 2) will commence with a pilot in December 2017. The trial will assess the effectiveness of both clarithromycin for 3 months and endoscopic sinus surgery.

# Hypothesis

By assessing biological and clinical parameters in patients with CRS, can we enable a clearer definition of potential disease endotypes and their relationship to the clinical phenotypes and subsequent treatment?

# Aims and objectives

The main goal of this work is to explore the endotypes in CRS. To achieve this, six main research goals will be addressed:

1. To explore the possibility of biological signatures using currently identified biomarkers (from the literature) in specific CRS patient groups that correlate with clinical parameters and outcomes in terms of response to treatment
2. To test the in vitro effect of clarithromycin on cytokine responses in epithelial cell cultures
3. To determine the effect of storage techniques on cytokine measurement and thus the necessary means of storage during the MACRO Programme Grant trial
4. To specify a core set of biomarkers that should be analysed further during the course of the MACRO Programme Grant trial including identification of any additional biological and clinical parameters that are not routinely collected
5. To collect specimens from subjects recruited to the MACRO trial for cytokine analysis to compare responders and non-responders to the interventions
6. To define the bacterial and fungal profiles of MACRO trial participants and investigate the microbial community stability after surgical or medical intervention

# Comparisons will also be made between control participants (without CRS) and CRS patients to establish whether any biomarker patterns identified are unique to CRS.

# Experimental design and methods

## Study design

### 1) Eligibility criteria

#### Inclusion:

Adult patients with a diagnosis of CRS according to European guidelines who have >12 week history of nasal congestion and/or nasal discharge along with hyposmia and/or facial pressure/pain and confirmation of disease on endoscopy and/or CT scan.

Control patients consist of patients aged 18 and above without a diagnosis of CRS and who undergo a septoplasty surgery for anatomical reasons.

#### Exclusion:

- Within 6 months post-operative
- Rare/complex sinus conditions
- CRS secondary to systemic disease such as cystic fibrosis and granulomatous disease suspected malignancy
- Pregnant/Lactating women
- Immunodeficiency states including HIV and selective and multiple antibody deficiency states
- Inability to give consent or to understand and comply with study instructions
- Control patients cannot suffer from nasal allergy.

### 2) Sampling and recruitment

A purposive sample of adult patients with CRS with or without polyps will be recruited both from the James Paget University Hospital and the Norfolk and Norwich University Hospital; other sites may subsequently be added through the NIHR CRN. The exact number of patients is difficult to predict in advance since sampling will continue until possible cytokine profiles emerge, however it is anticipated that it will be at least 50 individuals per group. Maximum variation sampling will guide recruitment and this will ensure a range of age, gender, ethnic background, location, previous treatments and symptom profile. Patients will be approached in clinic by the PI or the research nurse and will be given a recruitment pack that will include a participant information sheet, a consent form and a questionnaire. Patients who are interested will have their samples collected in clinic or in theatre. Samples will consist of a blood sample as well as nasal tissue and nasal mucus via swab. After collection, these samples will be transported to the Biorepository situated at the BCRE, UEA, Norwich.

For patients who already have tissue stored at the Biorepository, a retrospective recruitment will take place to seek approval to review clinical data. All these patients will be contacted, and if interested, a recruitment pack will be sent to them. All patients will be invited to complete an online questionnaire and provided with a unique secure PIN to access it.

### 3) Collection of samples

Samples will be collected in clinic or theatre.

Samples collected by ENT surgeon in theatre are handed to the surgical team, who will label them and keep them on ice until the end of the surgery. After the surgery, all samples will be stored at -20°C in JPUH or NNUH until transport is assured to the BCRE (Biorepository), situated in Norwich Research Park (UEA).

Transport to the BCRE will be assured by the researcher team and will be performed at regular intervals. Samples will be transported to the BCRE in a box filled with ice. Transportation will respect the RA transport form.

After reception at the BCRE, samples will be anonymised and stored at -80°C till analysis.

### 3) Sample Analysis

The collected samples will be stored at the Biorepository (UEA, Norwich) until the experimental analysis can take place. The laboratory analysis will consist of multiplex experiment, and will be conducted at the BMRC (UEA, Norwich). All these experiments will generate a large amount of data that will be correlated later on with the clinical data from the patients. The exact choice of biomarkers will be based on the literature search as well as prior experience and knowledge of chronic inflammatory conditions (2). IL-1β, IL-6, IL-8, IFN-γ , IL-17A, are pro-inflammatory cytokines generated predominately by macrophages and neutrophils, G-CSF IL-13, TGF-β affect T-helper cell polarisation and inhibit the production of pro-inflammatory cytokines, IL-5 stimulates B-cell growth and immunoglobulin secretion while IL-17A, IL-25, IL-33 are important for regulation of innate type II lymphoid cells which a have been implicated recently in allergy. MIP-1β, and GM-CSF stimulate recruitment of macrophages, while staphylococcal exotoxins-specific IgE and high polyclonal IgE levels are thought to be important precipitating factors of some cases of CRSwNPs. This combination of markers will allow us to stratify patients into those with predominantly pro-inflammatory phenotype (IL-1β, IL-6, IL-8, IFN-γ , IL-17A), possibly indicating macrophage-neutrophil involvement (MIP-1β, and GM-CSF), patients with changes in acquired T and B-cell meditated immune responses (eg: G-CSF IL-13, TGF-β) and those where disease may be underpinned by allergic reactions (eg: IgE, IL-17A, IL-25, IL-33). The nasal polyp tissue or nasal mucus will be processed by homogenization, centrifuging of suspensions followed by storage at -80°C until analysis for cytokines is performed. Supernatants will be assayed for the final list of cytokines using multiplex assays (Meso Scale). As it is possible that storage of the current samples in the biorepository may have an effect on the cytokine stability, this preliminary work will also compare the yield with freshly harvested tissue and help optimise the storage requirements within the trial. Using confluent epithelial monolayers from CRS patients’ fresh sinus epithelial tissue grown in vitro, a further analysis would be performed before and after exposure to clarithromycin^5^. The cytokine response between phenotypes (CRSwNPs, CRSsNPs) and between identified endotypes will be compared. Microbiome analysis would be undertaken in conjunction with a bioinformatics team led by Dr Falk Hildebrand at Quadram Institute Bioscience/Earlham Institute using established molecular microbiology protocols (16S amplicon sequencing using illumine miSeq).

### 4) Data analysis

The clinical data will be collated and correlated with the laboratory data and then analysed at UEA. The MACRO team will also review the results as part of the Programme team meetings. Analysis of electronic health records in Workstream 1 of the programme grant will also enable any modifications to the data collection required within the trial of both clinical and biological data to help define CRS endotypes further, ensuring the necessary resources are present and enable appropriate consent from trial participants. A control population will be sought from a parallel clinical trial of septoplasty where patients with CRS will be excluded. Further funding will be sought as appropriate for further work on the biological data collected during the trial including any additional techniques required (e.g. PCR, microarrays) and relevant consumable and staff costs. This will allow the work to be continued during the trial, allowing for comparison of the responders and non-responders in each of the 3 trial arms.

# Ethical approval

The NHS confirmed HRA approval on the 5th December 2016 under the reference 16/EM/0468.

# References

1. Hastan D, Fokkens WJ, Bachert C, et al. Chronic rhinosinusitis in Europe--an underestimated disease. A GA(2)LEN study. Allergy 2011;**66**(9):1216-23 doi: 10.1111/j.1398-9995.2011.02646.xpublished Online First: Epub Date]|.

2. Fokkens WJ, Lund VJ, Mullol J, et al. European Position Paper on Rhinosinusitis and Nasal Polyps 2012. Rhinol Suppl 2012(23):3 p preceding table of contents, 1-298

3. Thamboo A, Thamboo A, Philpott C, et al. Single-blind study of manuka honey in allergic fungal rhinosinusitis. J Otolaryngol Head Neck Surg 2011;**40**(3):238-43

4. Akdis CA, Bachert C, Cingi C, et al. Endotypes and phenotypes of chronic rhinosinusitis: a PRACTALL document of the European Academy of Allergy and Clinical Immunology and the American Academy of Allergy, Asthma & Immunology. J Allergy Clin Immunol 2013;**131**(6):1479-90 doi: 10.1016/j.jaci.2013.02.036published Online First: Epub Date]|.

5. Elmorsy S, El-Naggar MM, Abdel aal SM, et al. Sinus aspirates in chronic rhinosinusitis: fungal colonization of paranasal sinuses, evaluation of ICAM-1 and IL-8 and studying of immunological effect of long-term macrolide therapy. Rhinology 2010;**48**(3):312-7 doi: 10.4193/Rhin09.140published Online First: Epub Date]|.
