# Supplementary material for: Exploring Endotypes in Chronic Rhinosinusitis (ExpRess): Protocol for a cohort study
Source: PLoS One. 2023 Aug 2;18(8):e0289407. doi: 10.1371/journal.pone.0289407 (PMC10395813; doi:10.1371/journal.pone.0289407)
Supplement: S3 File — (PDF) [file pone.0289407.s004.pdf]

Mr Carl Philpott
Norwich Medical School, Chancellor's Drive
University of East Anglia, Norwich
Norfolk
NR4 7TJ

Email: hra.approval@nhs.net

05 December 2016

Dear Mr Philpott

Letter of HRA Approval

**Study title:**	**Exploring Endotypes in Chronic Rhinosinusitis (ExpRess)**
**IRAS project ID:**	**202585**
**REC reference:**	**16/EM/0468**
**Sponsor**	**University of East Anglia**

I am pleased to confirm that **HRA Approval** has been given for the above referenced study, on the basis described in the application form, protocol, supporting documentation and any clarifications noted in this letter.

Participation of NHS Organisations in England

The sponsor should now provide a copy of this letter to all participating NHS organisations in England.

Appendix B provides important information for sponsors and participating NHS organisations in England for arranging and confirming capacity and capability. **Please read *Appendix B* carefully**, in particular the following sections:

- *Participating NHS organisations in England* – this clarifies the types of participating organisations in the study and whether or not all organisations will be undertaking the same activities
- *Confirmation of capacity and capability* - this confirms whether or not each type of participating NHS organisation in England is expected to give formal confirmation of capacity and capability. Where formal confirmation is not expected, the section also provides details on the time limit given to participating organisations to opt out of the study, or request additional time, before their participation is assumed.
- *Allocation of responsibilities and rights are agreed and documented (4.1 of HRA assessment criteria)* - this provides detail on the form of agreement to be used in the study to confirm capacity and capability, where applicable.

Further information on funding, HR processes, and compliance with HRA criteria and standards is also provided.

It is critical that you involve both the research management function (e.g. R&D office) supporting each organisation and the local research team (where there is one) in setting up your study. Contact details

and further information about working with the research management function for each organisation can be accessed from www.hra.nhs.uk/hra-approval.

Appendices

The HRA Approval letter contains the following appendices:

- A – List of documents reviewed during HRA assessment
- B – Summary of HRA assessment

After HRA Approval

The document *"After Ethical Review – guidance for sponsors and investigators",* issued with your REC favourable opinion, gives detailed guidance on reporting expectations for studies, including:

- Registration of research
- Notifying amendments
- Notifying the end of the study

The HRA website also provides guidance on these topics, and is updated in the light of changes in reporting expectations or procedures.

In addition to the guidance in the above, please note the following:

- HRA Approval applies for the duration of your REC favourable opinion, unless otherwise notified in writing by the HRA.
- Substantial amendments should be submitted directly to the Research Ethics Committee, as detailed in the *After Ethical Review* document. Non-substantial amendments should be submitted for review by the HRA using the form provided on the HRA website, and emailed to hra.amendments@nhs.net.
- The HRA will categorise amendments (substantial and non-substantial) and issue confirmation of continued HRA Approval. Further details can be found on the HRA website.

Scope

HRA Approval provides an approval for research involving patients or staff in NHS organisations in England.

If your study involves NHS organisations in other countries in the UK, please contact the relevant national coordinating functions for support and advice. Further information can be found at http://www.hra.nhs.uk/resources/applying-for-reviews/nhs-hsc-rd-review/.

If there are participating non-NHS organisations, local agreement should be obtained in accordance with the procedures of the local participating non-NHS organisation.

User Feedback

The Health Research Authority is continually striving to provide a high quality service to all applicants and sponsors. You are invited to give your view of the service you have received and the application procedure. If you wish to make your views known please email the HRA at hra.approval@nhs.net. Additionally, one of our staff would be happy to call and discuss your experience of HRA Approval.

HRA Training

We are pleased to welcome researchers and research management staff at our training days – see details at http://www.hra.nhs.uk/hra-training/

Your IRAS project ID is **202585**. Please quote this on all correspondence.

Yours sincerely

Miss Lauren Allen

Assessor

Email: hra.approval@nhs.net

Copy to: *Mrs Yvonne Kirkham (Sponsor contact)*

Ms Joanne Lucas, James Paget University Hospital NHS Foundation Trust (Lead NHS R&D contact)

NIHR CRN Portfolio Applications Team

Appendix A - List of Documents

The final document set assessed and approved by HRA Approval is listed below.

Document	*Version*	*Date*
Contract/Study Agreement [HRA SoA]	1	23 November 2016
Contract/Study Agreement [MTA Human Tissue Schedule 2016]		
Evidence of Sponsor insurance or indemnity (non NHS Sponsors only)		
IRAS Application Form [IRAS_Form_28102016]		28 October 2016
Letter from funder [Letter from SJTT]		
Letter from sponsor [Indemnity from Sponsor]		
Other [Schedule of Events]	1	23 November 2016
Participant consent form	1.2	29 November 2016
Participant information sheet (PIS) [ExpRess Study PIS]	1.1	07 November 2016
Participant information sheet (PIS) [Control PIS]	1.1	07 November 2016
Summary CV for student [Student CV]		
Summary CV for supervisor (student research) [CMP CV]		
Summary CV for supervisor (student research)		

Appendix B - Summary of HRA Assessment

This appendix provides assurance to you, the sponsor and the NHS in England that the study, as reviewed for HRA Approval, is compliant with relevant standards. It also provides information and clarification, where appropriate, to participating NHS organisations in England to assist in assessing and arranging capacity and capability.

For information on how the sponsor should be working with participating NHS organisations in England, please refer to the, *participating NHS organisations*, *capacity and capability* and *Allocation of responsibilities and rights are agreed and documented (4.1 of HRA assessment criteria)* sections in this appendix.

The following person is the sponsor contact for the purpose of addressing participating organisation questions relating to the study:

Name: Mrs Yvonne Kirkham

Tel: 01603591721

Email: Y.Kirkham@uea.ac.uk

HRA assessment criteria

Section	HRA Assessment Criteria	Compliant with Standards	Comments
1.1	IRAS application completed correctly	Yes	No comments
2.1	Participant information/consent documents and consent process	Yes	A minor amendment was made to the consent form following REC favourable opinion to clarify that item 2 is not applicable for all participants.
3.1	Protocol assessment	Yes	No comments
4.1	Allocation of responsibilities and rights are agreed and documented	Yes	The Statement of Activities and Schedule of Events will act as the agreement between the sponsor and site.<br><br>A separate agreement has been provided for the transfer of samples.
4.2	Insurance/indemnity arrangements assessed	Yes	Where applicable, independent contractors (e.g. General Practitioners) should ensure that the professional indemnity provided by their medical defence organisation covers the activities expected of them for this research study
4.3	Financial arrangements assessed	Yes	No funding will be provided to the site.
5.1	Compliance with the Data Protection Act and data security issues assessed	Yes	No comments
5.2	CTIMPS – Arrangements for compliance with the Clinical Trials Regulations assessed	Not Applicable	No comments
5.3	Compliance with any applicable laws or regulations	Yes	The research will involve analysis of new and existing samples.

Section	HRA Assessment Criteria	Compliant with Standards	Comments
6.1	NHS Research Ethics Committee favourable opinion received for applicable studies	Yes	No comments
6.2	CTIMPS – Clinical Trials Authorisation (CTA) letter received	Not Applicable	No comments
6.3	Devices – MHRA notice of no objection received	Not Applicable	No comments
6.4	Other regulatory approvals and authorisations received	Not Applicable	No comments

Participating NHS Organisations in England

This provides detail on the types of participating NHS organisations in the study and a statement as to whether the activities at all organisations are the same or different.

There is one site type. The study activity will be conducted at the site as detailed in the protocol and supporting documentation.

The Chief Investigator or sponsor should share relevant study documents with participating NHS organisations in England in order to put arrangements in place to deliver the study. The documents should be sent to both the local study team, where applicable, and the office providing the research management function at the participating organisation. For NIHR CRN Portfolio studies, the Local LCRN contact should also be copied into this correspondence. For further guidance on working with participating NHS organisations please see the HRA website.

If chief investigators, sponsors or principal investigators are asked to complete site level forms for participating NHS organisations in England which are not provided in IRAS or on the HRA website, the chief investigator, sponsor or principal investigator should notify the HRA immediately at hra.approval@nhs.net. The HRA will work with these organisations to achieve a consistent approach to information provision.

Confirmation of Capacity and Capability

This describes whether formal confirmation of capacity and capability is expected from participating NHS organisations in England.

Participating NHS organisations in England **will be expected to formally confirm their capacity and capability to host this research**.

- Following issue of this letter, participating NHS organisations in England may now confirm to the sponsor their capacity and capability to host this research, when ready to do so. How capacity and capacity will be confirmed is detailed in the *Allocation of responsibilities and rights are agreed and documented (4.1 of HRA assessment criteria)* section of this appendix.

• The Assessing, Arranging, and Confirming document on the HRA website provides further information for the sponsor and NHS organisations on assessing, arranging and confirming capacity and capability.

Principal Investigator Suitability

This confirms whether the sponsor position on whether a PI, LC or neither should be in place is correct for each type of participating NHS organisation in England and the minimum expectations for education, training and experience that PIs should meet (where applicable).
The Chief Investigator will act as Principal Investigator at the site.<br><br>GCP training is not a generic training expectation, in line with the HRA statement on training expectations.

HR Good Practice Resource Pack Expectations

This confirms the HR Good Practice Resource Pack expectations for the study and the pre-engagement checks that should and should not be undertaken
No access arrangements will be needed for activity conducted by staff employed by site.<br><br>For the external research team from the University an Honorary Research Contract will be needed to undertake activity that will impact on patients' care (such as consent procedures and collection of samples) or a Letter of Access to undertake activity that will not directly impact on patients' care (such as collection of data from medical records). Disclosure and Barring Service and Occupational Health checks will be expected where an Honorary Research Contract is needed.<br>For staff employed by another Trust then NHS to NHS permissions will be needed.

Other Information to Aid Study Set-up

This details any other information that may be helpful to sponsors and participating NHS organisations in England to aid study set-up.
• The applicant has indicated that they intend to apply for inclusion on the NIHR CRN Portfolio.
